# Morphology of human sinoatrial node and its surrounding right atrial muscle in the global obesity pandemic—does fat matter?

**DOI:** 10.3389/fmed.2024.1415065

**Published:** 2024-06-19

**Authors:** Weixuan Chen, Daniel Rams, Maciej Zając, Raghad Albalawi, Andrew J. Atkinson, Abimbola J. Aminu, Malgorzata Mazur, Mateusz K. Hołda, Jerzy Walocha, Krzysztof Gil, Marcin Kuniewicz, Halina Dobrzynski

**Affiliations:** ^1^Division of Cardiovascular Sciences, School of Medical Sciences, University of Manchester, Manchester, United Kingdom; ^2^Department of Anatomy, Jagiellonian University, Kraków, Poland; ^3^Heart Embryology and Anatomy Research Team, Department of Anatomy, Jagiellonian University Medical College, Kraków, Poland; ^4^Department of Pathophysiology, Jagiellonian University Medical College, Kraków, Poland

**Keywords:** human sinus/sinoatrial node, right atrium, obesity, aging, fibrosis, cellular hypertrophy, P wave morphology

## Abstract

**Introduction:**

The sinus node (SN) is the main pacemaker site of the heart, located in the upper right atrium at the junction of the superior vena cava and right atrium. The precise morphology of the SN in the human heart remains relatively unclear especially the SN microscopical anatomy in the hearts of aged and obese individuals. In this study, the histology of the SN with surrounding right atrial (RA) muscle was analyzed from young non-obese, aged non-obese, aged obese and young obese individuals. The impacts of aging and obesity on fibrosis, apoptosis and cellular hypertrophy were investigated in the SN and RA. Moreover, the impact of obesity on P wave morphology in ECG was also analyzed to determine the speed and conduction of the impulse generated by the SN.

**Methods:**

Human SN/RA specimens were dissected from 23 post-mortem hearts (preserved in 4% formaldehyde solution), under Polish local ethical rules. The SN/RA tissue blocks were embedded in paraffin and histologically stained with Masson’s Trichrome. High and low-magnification images were taken, and analysis was done for appropriate statistical tests on Prism (GraphPad, USA). 12-lead ECGs from 14 patients under Polish local ethical rules were obtained. The P wave morphologies from lead II, lead III and lead aVF were analyzed.

**Results:**

Compared to the surrounding RA, the SN in all four groups has significantly more connective tissue (*P* ≤ 0.05) (young non-obese individuals, aged non-obese individuals, aged obese individuals and young obese individuals) and significantly smaller nodal cells (*P* ≤ 0.05) (young non-obese individuals, aged non-obese individuals, aged obese individuals, young obese individuals). In aging, overall, there was a significant increase in fibrosis, apoptosis, and cellular hypertrophy in the SN (*P* ≤ 0.05) and RA (*P* ≤ 0.05). Obesity did not further exacerbate fibrosis but caused a further increase in cellular hypertrophy (SN *P* ≤ 0.05, RA *P* ≤ 0.05), especially in young obese individuals. However, there was more infiltrating fat within the SN and RA bundles in obesity. Compared to the young non-obese individuals, the young obese individuals showed decreased P wave amplitude and P wave slope in aVF lead.

**Discussion:**

Aging and obesity are two risk factors for extensive fibrosis and cellular hypertrophy in SN and RA. Obesity exacerbates the morphological alterations, especially hypertrophy of nodal and atrial myocytes. These morphological alterations might lead to functional alterations and eventually cause cardiovascular diseases, such as SN dysfunction, atrial fibrillation, bradycardia, and heart failure.

## 1 Introduction

**FIGURE 1:**
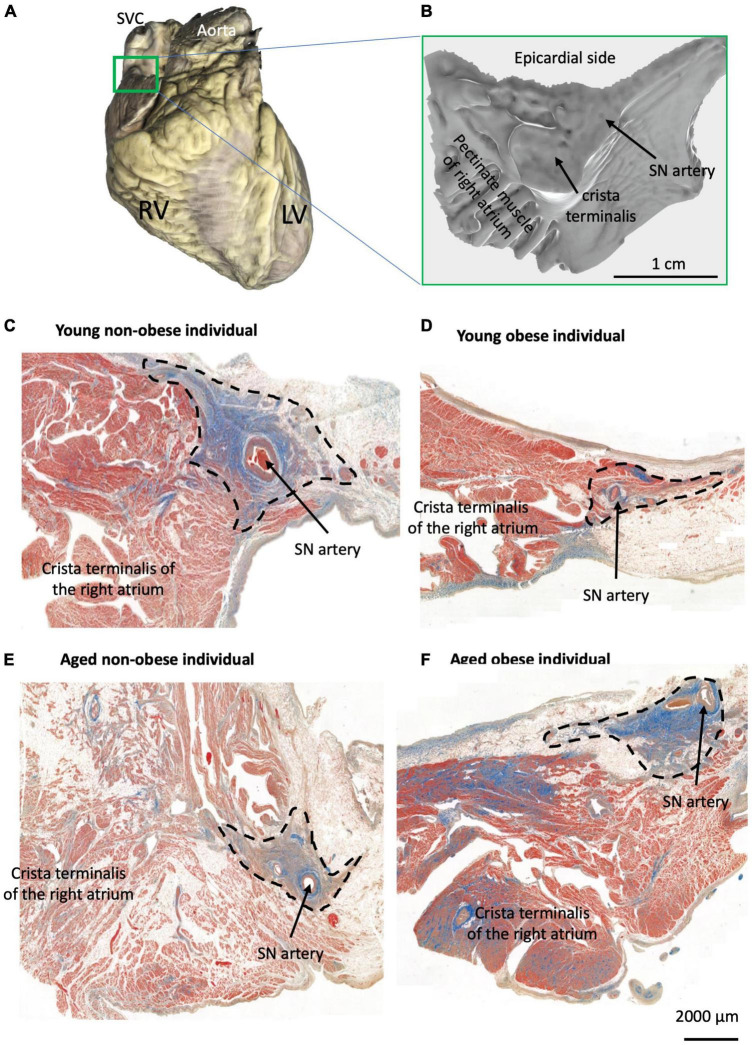
Identification of the SN region in four groups. **(A)** Example of one scanned whole heart with the SN/RA preparation indicated by the green rectangle from all hearts used for histology (total of 23 hearts). **(B)** Example of one scanned dissected SN/RA preparation. **(C–F)** Masson’s trichrome staining of the SN tissue sections and crista terminalis (the main branch of RA’s pectinate muscle) in one young non-obese individual **(C)**, one young obese individual **(D)**, one aged non-obese individual **(E)**, and one aged obese individual **(F)**. Blue = connective tissue, red = myocyte, pale white = fat.

The sinus node (SN) or sinoatrial node is a cluster of specialized cells called pacemaker cells that initiate and regulate heart rate. It lies at the junctional area between the superior vena cava and right atrium (Figures 1A, B), and travels along the crista terminalis toward the inferior vena cava (1, 2). The pacemaker cells or the myocyte of the SN are embedded in connective tissue and surround the distinct SN artery (Figure 1) (3). In general, the pacemaker cells in the SN are smaller than the myocytes in the myocardium. In humans, the diameter of the pacemaker cells is around 5–10 micrometers (μm), while the diameter of right atrial myocytes is around 15–20 μm (4, 5).

Obesity is one of the most concerned health issues worldwide. The World Health Organization (WHO) defines obesity as body mass index (BMI), the ratio of body mass to body height, equal to or larger than 30, and it is classified into three classes: BMI between 30 and 35 is Class I, BMI between 35 and 40 is Class II and BMI over 40 is Class III. Obesity is one of the main risk factors for various cardiovascular diseases and potential death. According to the World Obesity Atlas 2023, 14% of the world population over 5 years of age were classified as obese (BMI ≥ 30kg/m^2^) in 2020, and it is estimated to increase to 24% in 2035 (6). In 2015, 4 million deaths occurred due to high BMI, and more than two-thirds of these deaths were due to cardiovascular diseases (7). Some prevalent diseases related to obesity include hypertrophic cardiomyopathy, coronary artery disease, heart failure, atrial fibrillation, and sudden death (8, 9). The increased oxygen demands and blood volume that are commonly seen in obese populations could lead to chamber hypertrophy especially left ventricle hypertrophy and coronary artery diseases (9, 10). Moreover, neurohormonal activation, such as insulin resistance and abnormal glucose transport, could also lead to cardiac remodeling (8). It has also been shown previously that obese individuals tend to have increased fat surrounding the SN region, the so-called fatty infiltration (11). Obesity also causes increased bundle size, which means thicker pectinate muscles and crista terminalis (11).

According to WHO, aging is considered a natural biological process but involves a wide range of complex physical, mental, and social changes. In general, an adult older than 60 years of age would be considered an aged individual. Although it is a natural biological process, it is also one risk factor that significantly increases the risk of developing cardiovascular diseases. During cardiac aging, the cardiac function gradually decreases, the metabolic microenvironment deteriorates, and oxidative stress and mitochondrial abnormalities increase, which lead to fibrosis, hypertrophy, myocyte systolic dysfunction and vascular remodeling (12–14). Various animal studies showed that in the SN specifically, aging is associated with hypertrophy of nodal myocytes and enlargement of SN, which leads to a slow action potential in the centre, and this change in electrophysiology remodeled the SN and caused the decline in its function (15–18). Aged humans and dogs also tend to have increased connective tissue and decreased nodal cells that slow the heart rate, lower the overall cardiac output, and play a role in SN dysfunction (11, 19). These morphological changes might contribute to a higher incidence rate of arrhythmias in the aged population (18).

The electrocardiogram (ECG) is a diagnostic test commonly used in clinics. Aging and obesity are associated with many ECG alterations and abnormalities (20). In obesity, PR interval, QRS duration, and QTc interval all increased, and QRS voltage decreased, which shows a higher likelihood of alterations in ECG in the obese population (20–22). However, a recent clustering analysis shows that obesity does not strongly correlate with alterations and variations (23). Whilst in aging, it is more likely to show a decrease in QRS amplitude and T wave amplitude (24). The P wave amplitude is also decreased, the PR interval is prolonged, and the percentage of missing P wave is increased (25).

The purpose of this study was to investigate, illustrate and analyze human SN and its surrounding RA at the micro-anatomy level in normal/healthy, aged, and obese individuals and observe any functional alterations that can be detected in P wave morphology which could be associated with the micro-anatomical changes. We hypothesize that aging and/or obesity led to fibrosis, apoptosis, and cellular hypertrophy of the human SN as well as its surrounding RA muscle.

## 2 Materials and methods

### 2.1 Human sample details

For histology analysis, twenty-three *ex vivo* human hearts (13 males and 10 females); eight young non-obese hearts, six aged non-obese hearts, six aged obese hearts and three young obese hearts were used in this study (see Table 1 for details). Hearts were collected by the HEART - Heart Embryology and Anatomy Research Team, Department of Anatomy, Jagiellonian University Medical College, Krakow, Poland during routine forensic medical autopsies of individuals who died because of external causes (suicide, criminal acts, or accidents). Only hearts with no history of arrhythmia, no evidence of macroscopic cardiac pathologies, no past cardiac surgery, and no heart trauma were included in this study. Hearts were routinely dissected from the chest cavity. Subsequently, hearts were washed and fixed by passive immersion in the 4% formaldehyde solution.

**TABLE 1:** Patient information for histology analysis.

	Age (years)	Height (cm)	BMI (kg/m^2^)	BW (kg)	HW (g)	HW/BW (kg/kg)
Young non-obese group (*N* = 8 4M and 4F)	29.88 ± 2.36*‡	164.60 ± 3.24	23.46 ± 0.50¥‡	64.00 ± 3.60¥‡	316.10 ± 28.14¥‡	4.89E−3 ± 2.15E−4*
Young obese group (*N* = 3, 3M)	30.00 ± 4.51#†	170.70 ± 3.38	33.87 ± 2.74¥†	99.00 ± 10.54¥†	533.00 ± 46.65¥†	5.59E−3 ± 9.67E−4
Aged non-obese group (*N* = 6, 3M and 3F)	72.17 ± 3.50*†	166.70 ± 4.62	22.09 ± 0.69†§	61.67 ± 4.04†§	382.50 ± 37.40†§	6.24E−3 ± 5.10E−4*
Aged obese group (*N* = 6 3M, 3F)	64.33 ± 0.80#‡	164.20 ± 2.64	32.63 ± 0.93‡§	88.00 ± 3.59‡§	492.00 ± 30.76‡§	5.58E−3 ± 2.38E−4

In columns 2-7, mean ± SEM values are shown. N = number of specimens/hearts, M = male, F = female. The Two-way ANOVA tests were applied to test statistical significance. *P*-values less than 0.05 are shown in symbols.

* = statistical significance young non-obese vs. aged non-obese;

# = statistical significance young obese vs. aged obese;

† = statistical significance young obese vs. aged non-obese;

‡ = statistical significance young non-obese vs. aged obese;

¥ = statistical significance young non-obese vs. young obese;

§ = statistical significance aged non-obese vs. aged obese.

For ECG analysis, fourteen patients (8 males and 6 females with no antiarrhythmic treatment, no comorbidities with normal electrolyte levels, no inflammation or congenital heart defects) were recruited from the Department of Electrocardiology, John Paul II Hospital in Kraków, Poland. We used these two groups of patients because they did not take any antiarrhythmic medications (as older people commonly do) that could alter their ECG parameters.

This study was approved by the Bioethical Committee of the Jagiellonian University (No. 1072.6120.205.2019) and the Cardiology Clinic in Sw. Jana Pawla Hospital, Krakow, Poland (NB 060.1.005.2023). The study protocol conforms to the ethical guidelines of the 1975 Declaration of Helsinki.

### 2.2 Tissue block dissection and sectioning

All SN/RA tissue blocks were dissected by the experienced cardiac conduction system anatomist (Dr. Halina Dobrzynski) at the Department of Anatomy, Jagiellonian University Medical College, Krakow, Poland (Figures 1A, B) and as previously described by Chandler et al. (2). The SN regions with their surrounding atrial muscle were embedded in paraffin and serially sectioned at 5 μm (Figure 1B) in the Pathophysiology Department, Jagiellonian University Medical College, Krakow, Poland.

### 2.3 Masson’s trichrome staining and microscope imaging

The Masson’s trichrome staining was performed on 5 μm tissue sections from similar regions of the SN (closer to the region of the SVC in the RA, see Figures 1C–F). The detailed staining protocol can be referred to Kurnik-Łucka et al. (26) and Chandler et al. (2). The slides were stained in Celestine blue for 5 min, Mayer’s Alum haematoxylin for 10 min, acid fuchsin for 4 min, phosphomolybdic acid for 5 min and methyl blue for 5 min. Between each stain, the slides were washed in running tap water and distilled water for 30 min. The slides were then dehydrated in a series of ethanol, rinsed in histoclear for 5 min, and mounted in DPX before being covered in microscopic coverslips. After staining, the myocytes are stained pink/red/purple, connective tissue is stained blue, and fat cells appear white and hollow.

One tissue section per heart was scanned using a 3D-Histech Pannoramic-250 microscope slide scanner at 40x. After scanning, the software CaseViewer (3D Hishtech) was used to visualize the tissue section and take images of the SN and RA (Figures 1C–F). In this study, the region, that surrounds the SN artery and is packed with connective tissue was defined as the SN region, and the region in the crista terminalis and pectinate muscle was defined as the RA region (Figure 1). This region is well established in the published literature to be the SN region, within the RA, as previously shown by our group (1, 2, 11, 27–30).

**FIGURE 2:**
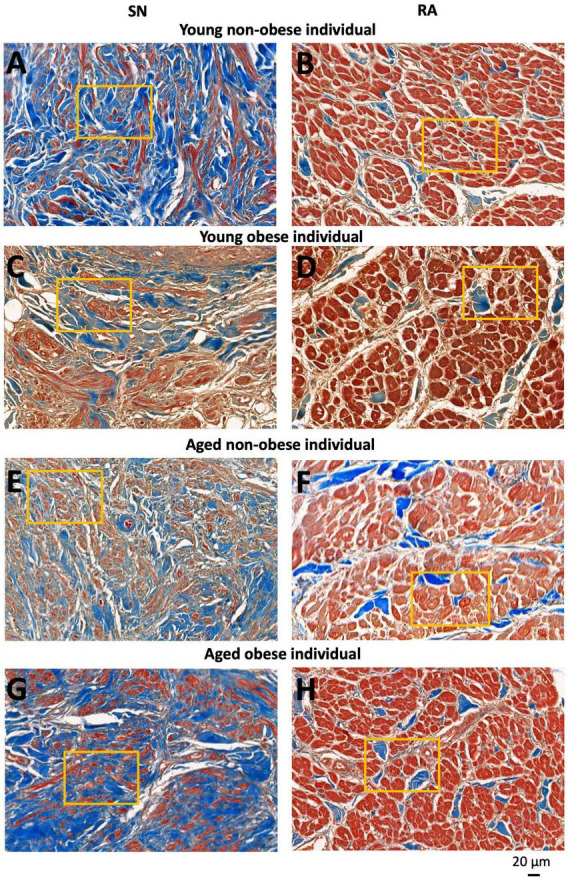
Representative high magnification images of Masson’s trichrome stained SN region (around the SN artery) and RA muscle (within the crista terminals region and/or pectinate muscle) of four groups investigated. SN and RA regions from the young non-obese group **(A,B)**, young obese group **(C,D)** aged non-obese group **(E,F)** and aged obese group **(G,H)**. The yellow rectangles indicate regions of closer views of the SN and RA shown in Figure 3. Blue = connective tissue, red = myocyte, pale white = fat.

**FIGURE 3:**
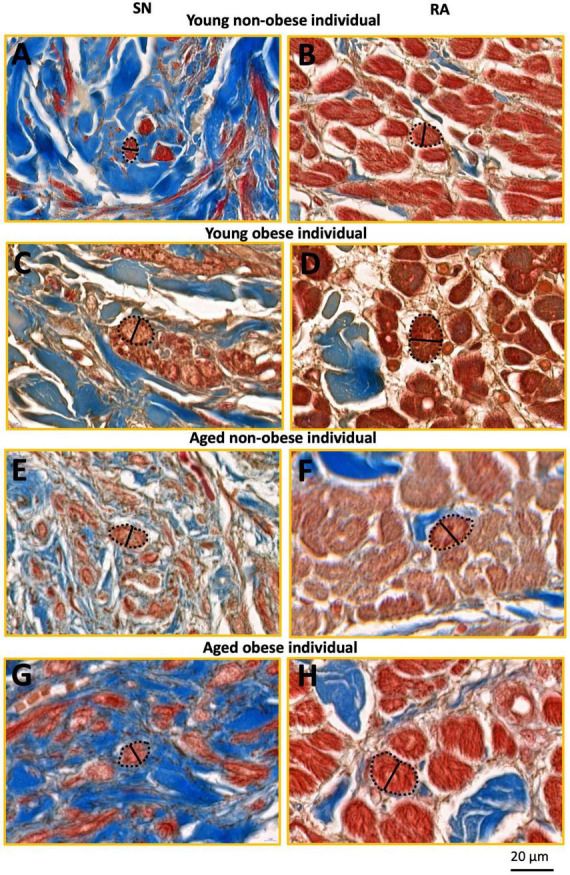
This figure shows inserts from Figure 2 (yellow rectangles). SN and RA regions from the young non-obese group **(A,B)**, young obese group **(C,D)**, aged non-obese group **(E,F)**, and aged obese group **(G,H)**. The dashed black lines circled the exampled myocytes being measured, and the black straight lines indicate the exampled measurements of the myocyte diameter in the SN and the RA. Blue = connective tissue, red = myocyte, pale white = fat.

For each tissue section and each tissue type (the SN and RA), three to four images were taken at the same magnification for further analysis of fibrosis, apoptosis, and cellular hypertrophy (Figures 2, 3). In total, for the young non-obese group, 31 images for the SN (Figures 2A, B) and 31 images for the RA were taken; for the young obese group, 10 images were taken for the SN (Figures 2C, D) and 10 images were taken for the RA; for the aged non-obese group, 23 images were taken for the SN (Figures 2E, F) and 23 images were taken for the RA; for the aged obese group, 23 images for the SN and 24 images were taken for the RA (Figures 2G, H).

The yellow rectangles in Figures 2, 3 are just for illustrative purpose for this paper. The myocytes throughout the images were randomly selected in ‘a blind’ manner for the students (who did not know the group names and heart numbers) to carry out measurements and statistical analysis. In this study, we tried to stay unbiased throughout the measurements and analysis described in Sections 2.4 and 2.5.

### 2.4 Imaging analysis for connective tissue and myocyte volume measurements

The images were analyzed using Image J^1^ for semi-quantitative analysis in this part of the study. To measure the area of the selected tissue type (myocyte or connective tissue), the “color threshold” in Image J was altered to ensure only one tissue type was highlighted. Then, the area of the selected tissue was measured and recorded on an Excel file. The % content of myocyte ($\frac{a\,r\,e\,a\,o\,f\,m\,y\,o\,c\,y\,t\,e}{a\,r\,e\,a\,o\,f\,m\,y\,o\,c\,y\,t\,e+a\,r\,e\,a\,o\,f\,c\,o\,n\,n\,e\,c\,t\,i\,v\,e\,t\,i\,s\,s\,u\,e}$), and % content of connective tissue ($\frac{a\,r\,e\,a\,o\,f\,c\,o\,n\,n\,e\,c\,t\,i\,v\,e\,t\,i\,s\,s\,u\,e}{a\,r\,e\,a\,o\,f\,m\,y\,o\,c\,y\,t\,e+a\,r\,e\,a\,o\,f\,c\,o\,n\,n\,e\,c\,t\,i\,v\,e\,t\,i\,s\,s\,u\,e}$) and the connective tissue/myocyte ratio ($\frac{a\,r\,e\,a\,o\,f\,c\,o\,n\,n\,e\,c\,t\,i\,v\,e\,t\,i\,s\,s\,u\,e}{a\,r\,e\,a\,o\,f\,m\,y\,o\,c\,y\,t\,e}$) were calculated in Excel and the statistical analysis were done in GraphPad Prism 10. All measurements were calculated and shown in Table 2.

**TABLE 2:** Summary of different measurements and analyses compared among four groups.

	Young non-obese group	Young obese group	Aged non-obese group	Aged obese group
Connective tissue/myocyte ratio (SN)	1.202 ± 0.169‡	1.920 ± 0.499	1.859 ± 0.255	2.301 ± 0.382‡
Connective tissue/myocyte ratio (RA)	0.107 ± 0.011*¥‡	0.304 ± 0.075¥†#	0.592 ± 0.048*†	0.611 ± 0.066‡#
Connective tissue % content (SN)	51.760 ± 3.296‡	62.680 ± 4.707	60.580 ± 3.157	65.440 ± 3.383‡
Connective tissue % content (RA)	9.479 ± 0.881*¥‡	28.370 ± 1.392¥†#	36.180 ± 1.765*†	36.170 ± 2.650‡#
Myocyte % content (SN)	48.240 ± 3.296‡	37.320 ± 4.707	39.420 ± 3.157	34.560 ± 3.383‡
Myocyte % content (RA)	90.520 ± 0.881*¥‡	71.630 ± 1.392¥†#	63.820 ± 1.765*†	63.830 ± 2.650‡#
Cell diameter (μm) (SN)	8.830 ± 0.206 (n = 83)*¥‡	13.840 ± 0.410 (n = 39)¥†#	12.350 ± 0.443 (n = 68)*†	11.610 ± 0.284 (n = 81)‡#
Cell diameter (μm) (RA)	15.100 ± 0.205 (n = 155)*¥‡	16.060 ± 0.408 (n = 60)¥†#	17.120 ± 0.216 (n = 115)*†§	18.260 ± 0.301 (n = 101)‡#§

In columns 2-5, mean ± SEM are shown. The Two-way ANOVA test was applied to test statistical significance. P values less than 0.05 are shown with symbols.

* = statistical significance young non-obese vs. aged non-obese group;

# = statistical significance young obese vs. aged obese group;

† = statistical significance young obese vs. aged non-obese group;

‡ = statistical significance young non-obese vs. aged obese group;

¥ = statistical significance young non-obese vs. young obese group;

§ = statistical significance aged non-obese vs. aged obese group.

### 2.5 Imaging analysis for cell diameter measurements

For each image taken from CaseViewer, 4-7 transverse and round cells were identified and measured. In summary, for the young non-obese group, 83 SN cells and 155 RA cells were measured (Figures 3A, B); for the young obese group, 39 SN cells and 60 RA cells were measured (Figures 3C, D); for the aged non-obese group, 68 SN cells and 115 RA cells were measured (Figures 3E, F); for the aged obese group, 81 SN cells and 101 RA cells were measured (Figures 3G, H). The ‘line measurement’ in Image J was used for cell diameter measurements. The cell diameter measurements in μm were calculated and shown in Table 2.

It has to be noted that most SN cells form interweaving arrangements with the connective tissue that they are embedded in (e.g., see Figures 2A, C, E, G), therefore these types of cells are difficult to measure because no cell boundaries can be easily obtained. We used only those nodal cells that are sliced in more transverse plane (as indicated in Figures 3A, C, E, G from the yellow boxes in Figures 2A, C, E, G) and in which clear cells diameter can be obtained. For comparison we selected atrial cells in a similar cut plane for measuring their diameter (Figures 3B, D, F, H). We think that this is the best wat to illustrate our semi-quantitative approach to cellular hypertrophy.

### 2.6 ECG analysis

**FIGURE 4:**
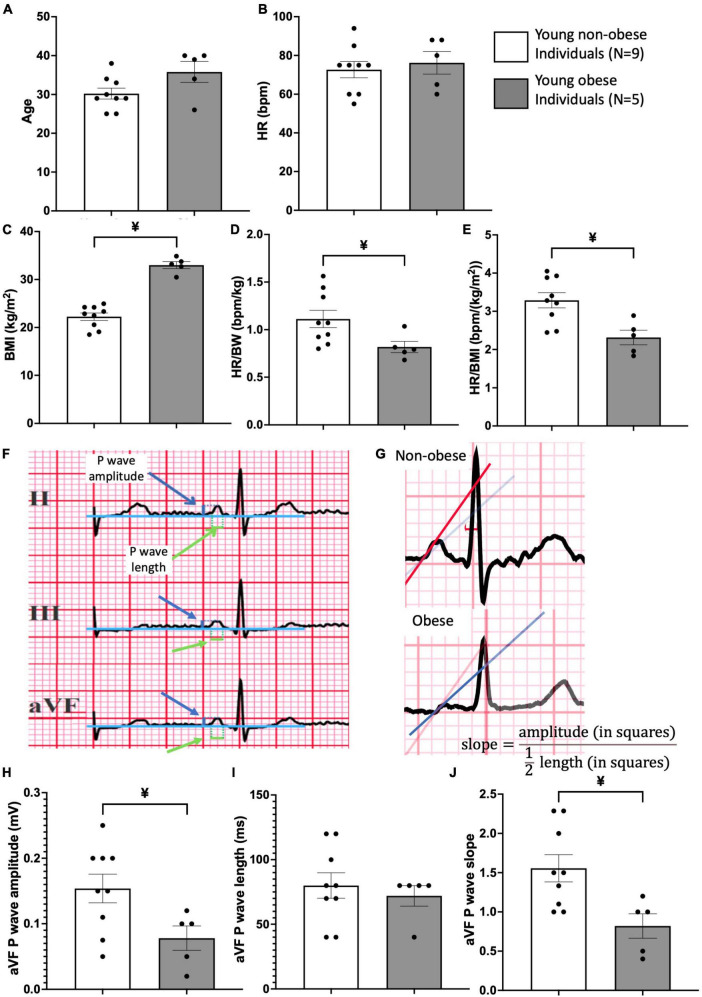
Patient information and ECG analysis of P wave morphology in young obese group vs. young non-obese group. Patient information on age **(A)**, heart rate (HR) **(B)**, Body Mass Index (BMI) **(C)**, HR/body weight ratio **(D)** and HR/BMI ratio **(E)** is provided. An example of the method used for measurements of P wave amplitude and length is shown **(F)** in leads II, III and aVF. Blue arrows point to P wave amplitude and green arrows point to P wave length. An example of the method used for P wave slope in non-obese and obese hearts is shown in panel **(G)**. Comparison of P wave amplitude **(H)**, length **(I)**, and slope **(J)** between the young non-obese and young obese group is provided from the aVF lead. Unpaired *t*-tests were done. ¥ = statistic significant young non-obese vs. young obese, *P*-values less than 0.05.

For all patients (Figure 4), the cardiac axis was within the nomogram range (+15 to +75 degrees), without intraventricular conduction disturbances or bundle branch blocks. The patients were allocated into two groups: non-obese and obese (Figure 4). The ECG recordings from all patients were attained, heart rate (HR), and P wave amplitude, P wavelength and P wave slope for leads II, III, aVF were assessed (Figures 4F, G). Three consecutive P waves were measured for both length and amplitude, and subsequently, the average values of those measurements were used in the formula for the slope, $s\,l\,o\,p\,e=\frac{a\,m\,p\,l\,i\,t\,u\,d\,e}{\frac{1}{2}\,l\,e\,n\,g\,t\,h}$. The leads II, III and aVF were chosen due to their similar orientations (facing downward).

### 2.7 Statistical analysis

Two-way ANOVA and unpaired t-tests were used for statistical analysis. GraphPad Prism 10 was used for producing graphs and statistical analysis. For the two groups’ comparison, unpaired t-tests were performed, and the statistical significance was indicated by different symbols (Figure 4 and Table 3). For comparisons of four groups, two-way ANOVA tests were performed and shown in Tables 1, 2.

**TABLE 3:** Summary of measurements and analyses comparing aging and obesity.

	Young vs. aged groups		Non-obese vs. obese groups	
	Young	Aged	Non-obese	Obese
Connective tissue/myocyte ratio (SN)	1.398 ± 0.1957$	2.080 ± 0.2287$	1.483 ± 0.1660€	2.174 ± 0.2925€
Connective tissue/myocyte ratio (RA)	0.1072 ± 0.01082$	0.3475 ± 0.08182$	0.3148 ± 0.06956	0.5085 ± 0.06985
Connective tissue % content (SN)	54.74 ± 3.017$	63.01 ± 2.324$	55.54 ± 2.540€	64.52 ± 2.613€
Connective tissue % content (RA)	14.63 ± 2.752$	36.18 ± 1.518$	20.92 ± 3.767€	33.57 ± 2.186€
Myocyte % content (SN)	45.26 ± 3.017$	36.99 ± 2.324$	44.46 ± 2.540€	35.48 ± 2.613€
Myocyte % content (RA)	85.37 ± 2.752$	63.82 ± 1.518$	79.08 ± 3.767€	66.43 ± 2.186€
Cell diameter (μm) (SN)	10.43 ± 0.2856$	11.95 ± 0.2554$	10.41 ± 0.2697€	12.34 ± 0.2515€
Cell diameter (μm) (RA)	15.37 ± 0.1884$	17.65 ± 0.1853$	15.96 ± 0.1613€	17.44 ± 0.2555€

The Unpaired *t*-test was applied to test statistical significance. *P*-values less than 0.05 are shown using symbols.

$ = statistical significance young vs. aged;

€ = statistical significance non-obese vs. obese.

## 3 Results

### 3.1 Patient clinical profile

The basic clinical data of the patients were obtained from the Jagiellonian University, Krakow, Poland. The heart weight (HW), body weight (BW), BMI, age, and HW to BW ratio of different groups were Tabulated in Table 1. The two obese groups show larger HW, BW and BMI values compared to the two non-obese groups of the same age (Table 1). The two aged groups show significantly increased age compared to the two young groups (Table 1). For the HW/BW ratio, there is a significant increase in the aged non-obese group compared to the young non-obese group.

### 3.2 Fibrosis and apoptosis in the SN and surrounding RA: connective tissue and myocyte contents

In this paper, the architecture of the human SN is described. In the young non-obese group, the SN can be identified by distinct increased connective tissues that surround the SN artery and it is located close to the crista terminalis (Figure 1C). In Figures 1D–F, the SN examples of the young obese group, aged non-obese group and aged obese group show increased fat tissue surrounding the SN region. Also, in the aged obese group (Figure 1F), more connective tissues can be identified in the SN, crista terminalis and other RA regions.

If closely observing the SN (Figures 2A, C, E, G) and RA regions (Figures 2B, D, F, H) all four groups show a similar trend that the SN has a significantly higher connective tissue (stained blue) to myocytes (stained red) ratio compared to the RA. The connective tissue to myocyte ratio in the SN was compared among four groups. It shows a complex situation. The aged obese group has a significantly higher connective tissue to myocyte ratio content than the young non-obese group (Table 2). The connective tissue to myocyte ratio in the RA was also compared among four groups (Table 2). There is a significant increase of connective tissue in two aged groups compared to young groups, and the young obese group compared to the young non-obese group, but there is no significant difference between the two aged groups.

Table 2 also shows the percentage of myocyte (stained in red) and connective tissue (stained in blue) in the SN and RA across four groups. In the SN, aged obese hearts show significantly decreased myocyte content compared to the young non-obese group. Compared to the young non-obese group in the RA, aged non-obese hearts, aged obese hearts, and young obese hearts have significantly decreased percentages of myocyte content.

### 3.3 Cellular hypertrophy of nodal and atrial myocytes

The width of the SN and RA myocytes were measured in young non-obese, young obese, aged non-obese, and aged obese groups (Figure 3). In all four groups, the atrial myocytes (Figures 3B, D, F, H) have significantly larger cellular diameters than nodal myocytes (Figures 3A, C, E, G).

The myocyte diameter in the SN region was compared among four groups (Table 2). The increased SN cell diameter can be observed in the aged non-obese group, young obese group and aged obese group compared to the young non-obese group. The young obese group also shows increased cell diameter than the aged obese group. The two aged groups have similar cell diameters, while the young obese group has the largest cell diameter. Overall, obesity plays a more important role than the aging factor (Table 3).

In the RA, the two aged groups have larger cellular diameters than the young groups. In addition, the young obese group has a larger cell diameter compared to the young non-obese group. Both aging and obesity contribute to cellular hypertrophy in RA, while aging plays a more important role (Tables 2, 3).

### 3.4 P wave morphology in young obese vs. young non-obese group

The SN and surrounding RA morphology were also compared between non-obese and obese groups without the aging factor. In the SN, the obese group has significantly increased connective tissue to myocyte ratio, decreased myocyte content, increased connective tissue content and larger myocyte diameter compared to the non-obese group (Table 3). In the RA, the obese group also shows decreased myocyte content, increased connective tissue content and larger myocyte diameter (Table 3).

These significant differences show the distinct morphological alterations seen in the obese group that lead to the ECG analysis to study the potential functional alterations in P wave morphology in obese young people. The rationale was the fact that we were able to obtain ECG recordings from young non-obese and young obese individuals who did not have any medications prescribed or suffered from any medically diagnosed heart and other conditions. We wanted to show a comparative morphological substrate from young non-obese vs. young obese individuals that can explain changes in the P wave morphology. This would not be possible for aged groups with complex medications and clinical conditions.

The ECG from two groups of patients were retrieved and compared. These patients were in a similar age group and the heart rate (HR) was similar (Figures 4A, B). The young obese group shows a larger BMI, and a significantly lower HR to body weight ratio and HR to BMI ratio compared to the young non-obese group, which indicates the conduction alterations in obesity (Figures 4C–E). From the P wave morphology analysis, aVF lead shows significant differences between the young non-obese and young obese groups. The young non-obese group has higher P wave amplitude and steeper P wave slope compared to the young obese group (Figures 4F–J).

## 4 Discussion

In the current study, we showed that in aging and obesity, there was extensive fibrosis and cellular hypertrophy in the SN and RA. Furthermore, obesity exacerbates the morphological alterations, namely hypertrophy of nodal and atrial myocytes.

This study investigated the micro-anatomy of SN and RA in young and healthy individuals. It confirms the finding from previous studies in humans and rodents that the SN is located close to the crista terminalis surrounds the SN artery, and is packed with an extensive amount of connective tissue (1–3, 11, 17, 27–30).

Both the aged group and obese group show a significant increase in connective tissue content and a decrease in myocyte content in the SN (Figure 2 and Tables 2, 3). This data aligns with previous human and canine research that in aging, increased fibrosis can be seen in the SN (11, 19). Further to the impact of aging, obesity can also lead to increased fibrosis and apoptosis in the human SN.

In the RA, all four groups show a small amount of connective tissue compared to the myocyte (Figures 2, 3). However, the two aged groups have a significantly higher connective tissue to myocyte ratio compared to the young groups, which indicates that aging is the main factor in RA fibrosis (Table 2). This corresponds with the previous canine studies that aging increases fibrosis in the myocardium (19). The young obese group shows increased connective tissue to myocyte ratio compared to the young non-obese group (Table 2). This shows that although aging is the main factor, obesity, especially obesity at a younger age can lead to micro-anatomical alterations in the RA.

An increase in fibrosis in the SN and its surrounding myocardium can be functionally problematic and produce an insulating border that delays the electrical impulses, which can lead to SN bradycardia. Furthermore, the insulating border can also lead to re-entry circuits around the SN that cause SN atrial tachycardia, and the insulating border in the RA may lead to atrial fibrillation (18, 31, 32).

The myocyte diameter in the SN is smaller than in the RA in all four groups (Figure 3 and Table 2). This corresponds to the previous study on various mammals including humans that the pacemaker cells are generally smaller than the myocytes in the myocardium (2, 4, 5).

Figure 3 shows that the two aged groups have larger pacemaker cell diameters than the cells in the young non-obese group, which indicates that aging plays a role in the SN cellular hypertrophy (Tables 2, 3). The SN in the young obese group has the largest cell diameter among the four groups, which means that obesity, especially obesity at a young age plays an important role in SN cellular hypertrophy (Table 2).

In the RA, the aged non-obese group has a larger cell diameter than the cells in the young non-obese group, and the aged obese group has a larger cell diameter than the cells in the young obese group (Figure 3 and Table 2). These two comparisons indicate that aging plays an important role in cellular hypertrophy in the RA, which aligns with previous research on humans that an aged heart has accumulated ROS and lipids, impairment of mitochondrial integrity and alteration of glucose metabolism from glucose oxidation to anaerobic glycolysis, and ultimately leads to cellular hypertrophy in the myocardium that impacts the contractile function (13, 33).

There is also a significant increase in the cell diameter in the young obese group compared to the young non-obese group and the aged obese group to the aged non-obese group (Figure 3 and Tables 2, 3). These comparisons suggest that obesity can also lead to cellular hypertrophy, and obesity exacerbates the impact that was made by aging. The cellular hypertrophy that can be seen in aging and obese humans indicates the increased likelihood of developing cardiac hypertrophy and cardiomyopathies (18, 34).

The hypertrophy also correlates with papillary muscle hypertrophy and cellular apoptosis, which leads to obstruction, ischemia and infarction that are commonly seen in hypertrophic cardiomyopathy in humans (11, 35).

The anatomical alterations seen in the obese SN impact the SN function which can be detected from 12-lead ECG. Since the SN is the main pacemaker site, it initiates the atrial depolarisation and produces the small P wave. As a result, any dimensional change on the P wave would indicate the functional change of the SN (36). Figures 4F–H shows that the aVF lead in young obese patients exhibits a significant decrease in P wave amplitude and P wave slope. P wave morphology alterations indicate the alterations in conduction, especially the functional alterations of SN. In human obesity, the increased fat content (see Figures 1D–F) releases an increased number of chemokines and inflammatory cytokines that can change the atrial myocardium and the SN through paracrine interactions (37, 38).

The increased fibrosis, myocyte apoptosis and cellular hypertrophy in obese patients can change the function of the SN. The altered P wave morphology and altered geometry of the structure indicate the SN and atrial dysfunction (38–40).

This is the first study that investigates the human SN and RA morphology in obese and aged individuals, differentiates the impact of obesity and aging on the SN and RA, and identifies the combined effects of these two factors. This study shows the importance of aging and obesity on the SN and RA morphology, especially for the young obese population. However, there are also some limitations. Due to the limited availability of the precious human sample, the young obese group has only three hearts, and all of them are males, while the other groups have combined sexes. Nevertheless, the three young obese hearts allowed us to do the statistical comparisons and semi-quantitative analysis. In the ECG analysis, we only used young non-obese and young obese groups to analyze the impact of obesity as explained in Section 3.4.

## Data availability statement

The original contributions presented in this study are included in this article/supplementary material, further inquiries can be directed to the corresponding author.

## Ethics statement

The studies involving humans were approved by the Bioethical Committee of the Jagiellonian University (No. 1072.6120.205.2019), the Cardiology Clinic in Sw. Jana Pawla Hospital, Krakow, Poland (NB 060.1.005.2023), and the ethical guidelines of the 1975 Declaration of Helsinki. The studies were conducted in accordance with the local legislation and institutional requirements. The ethics committee/institutional review board waived the requirement of written informed consent for participation from the participants or the participants’ legal guardians/next of kin because patients were informed that their ECG data would be used for research. Local Polish ethics allows the usage of anonymous patient data for research and the Polish local legal system allows the usage of post-portem tissue for research without relatives’ consent.

## Author contributions

WC: Writing – review and editing, Writing – original draft, Visualization, Supervision, Software, Methodology, Investigation, Formal analysis, Data curation. DR: Writing – review and editing, Software, Methodology, Formal analysis. MZ: Writing – review and editing, Validation, Software, Methodology, Formal analysis. RA: Writing – review and editing, Validation, Methodology, Formal analysis. AnA: Writing – review and editing, Supervision. AbA: Writing – review and editing, Supervision. MM: Writing – review and editing, Data curation. MH: Writing – review and editing, Supervision, Resources, Funding acquisition. JW: Writing – review and editing, Conceptualization. KG: Writing – review and editing, Supervision. MK: Writing – review and editing, Supervision, Resources, Conceptualization. HD: Writing – review and editing, Writing – original draft, Supervision, Resources, Project administration, Funding acquisition, Conceptualization.

## Footnotes

1
https://imagej.net/ij/
